# BOS1 is a basic helix–loop–helix transcription factor involved in regulating panicle development in rice

**DOI:** 10.3389/fpls.2023.1162828

**Published:** 2023-04-26

**Authors:** Yanpeng Lv, Xinfeng Zhang, Yanjuan Hu, Shuang Liu, Yanbin Yin, Xiaoxue Wang

**Affiliations:** Rice Research Institute, Shenyang Agricultural University, Shenyang, China

**Keywords:** BOS1, bos1-1, bHLH transcription factor, panicle development, panicle branch, rice

## Abstract

Panicle development is crucial to increase the grain yield of rice (*Oryza sativa*). The molecular mechanisms of the control of panicle development in rice remain unclear. In this study, we identified a mutant with abnormal panicles, termed *branch one seed 1-1* (*bos1-1*). The *bos1-1* mutant showed pleiotropic defects in panicle development, such as the abortion of lateral spikelets and the decreased number of primary panicle branches and secondary panicle branches. A combined map-based cloning and MutMap approach was used to clone *BOS1* gene. The *bos1-1* mutation was located in chromosome 1. A T-to-A mutation in *BOS1* was identified, which changed the codon from TAC to AAC, resulting in the amino acid change from tyrosine to asparagine. *BOS1* gene encoded a grass-specific basic helix–loop–helix transcription factor, which is a novel allele of the previously cloned *LAX PANICLE 1* (*LAX1*) gene. Spatial and temporal expression profile analyses showed that *BOS1* was expressed in young panicles and was induced by phytohormones. BOS1 protein was mainly localized in the nucleus. The expression of panicle development-related genes, such as *OsPIN2*, *OsPIN3*, *APO1*, and *FZP*, was changed by *bos1-1* mutation, suggesting that the genes may be the direct or indirect targets of BOS1 to regulate panicle development. The analysis of *BOS1* genomic variation, haplotype, and haplotype network showed that *BOS1* gene had several genomic variations and haplotypes. These results laid the foundation for us to further dissect the functions of BOS1.

## 1 Introduction

Rice (*Oryza sativa*) is a major food crop feeding more than half of the world’s population (Yoshida and Nagato, 2011; Zeng et al., 2017). With the increase in population and the decrease in arable land, improving rice productivity has become the main goal of breeding (Tilman et al., 2011; Zeng et al., 2017). Rice yield is a complex agronomic trait, which is mainly determined by three traits, such as panicle number, grain number per panicle, and grain weight (Xing and Zhang, 2010). These suggest that panicle development is very important to improve rice yield.

The shoot architecture of plants is dependent on the functions of shoot apical meristem (SAM) and axillary meristems (AMs) (Mcsteen and Leyser, 2005; Wang et al., 2018). In rice, SAM produces the main shoot, while AMs produce new shoot branches called tillers. The main culm and branches determine the biomass and the number of inflorescences and panicles in rice. Inflorescences in rice belong to the raceme class, with long branches called panicles. After the transition from the vegetative to reproductive phase in rice, SAM is changed into an inflorescence meristem (IM; rachis meristem). IM develops into the primary inflorescence axis (rachis) and initiates the primary branch meristem (PBM). PBM generates secondary branch meristem (SBM). At the top of the primary branches (PBs) and secondary branches (SBs), PBMs and SBMs are transformed into spikelet meristem (SM), resulting in terminal spikelets and flowers (Komatsu M.et al., 2003; Itoh et al., 2005; Yoshida et al., 2012). The determination of meristem fate greatly affects the size, morphology of rice panicles, and rice yield.

The transition from SAM to IM is controlled by heading date-related genes. The rice floral transition is promoted by florigens, such as Heading date 3a (Hd3a) and Rice Flowering Locus T 1 (RFT1) (Tsuji et al., 2013; Zhou S. et al., 2021; Zhao et al., 2022). In the SAM, Hd3a and RFT1 are physically associated with bZIP transcription factor OsFD1 and 14-3-3, a member of the Gf14 family, forming florigen activation complexes (FACs). FACs trigger the expression of floral identity genes, such as *OsMADS14*, *OsMADS15*, *OsMADS18*, and *OsMADS34*, to accelerate floral transition (Tamaki et al., 2007; Komiya et al., 2008; Taoka et al., 2011; Kobayashi et al., 2012). OsMADS34, also called Panicle Phytomer 2 (PAP2), is a member of the grass-specific SEPALLATA (SEP) clade of MADS-box proteins. *OsMADS14*, *OsMADS15*, and *OsMADS18* are the members of the APETALA 1 (AP1)/FRUITFULL (FUL) family (Zahn et al., 2005; Gao et al., 2010).

Axillary meristems generate panicle branches and florets in rice. Plant hormones, such as auxin and cytokinin (CK), and transcription factors are involved in axillary meristem initiation and outgrowth to control panicle branching in rice (Zhang and Yuan, 2014). Tryptophan Deficient Dwarf 1 (TDD1), an anthranilate synthase beta-subunit, catalyzes the first step of the tryptophan (Trp) biosynthesis and is involved in auxin biosynthesis in rice. The *tdd1* mutant shows pleiotropic phenotypes, such as deficiency in Trp and indole-3-acetic acid (IAA), dwarfing, narrow leaves, short roots, and abnormal flowers (Sazuka et al., 2009). *Grain number 1a* (*Gn1a*) encodes cytokinin oxidase/dehydrogenase (OsCKX2), which has the enzyme activity to degrade the cytokinin. Downregulated expression of *OsCKX2* causes cytokinin accumulation in IMs and increases the number of reproductive organs, resulting in enhanced grain yield (Ashikari et al., 2005). Drought and salt tolerance (DST), a rice zinc-finger transcription factor, directly upregulated the expression of *OsCKX2* in the reproductive meristem of rice (Zhang and Yuan, 2014). DST-dependent expression of *OsCKX2* regulates CK accumulation in the SAM to control the number of reproductive organs, such as panicle branching and grain number (Li et al., 2013).

Rice Aberrant Spikelet and Panicle 1 (ASP1), a transcriptional corepressor, plays important roles in auxin-related panicle development. The *asp1* mutant exhibits abnormal spikelets, which reduced the number of branch and spikelet phenotypes (Yoshida et al., 2012). A plant-specific transcription factor containing a conserved SBP-box DNA-binding domain Squamosa Promoter Binding Protein-Like 14 (OsSPL14), which is also known as Ideal Plant Architecture 1 (IPA1) and Wealthy Farmer’s Panicle (WFP), is involved in panicle branching (Yamasaki et al., 2004; Jiao et al., 2010; Miura et al., 2010). Increasing the *OsSPL14* expression in the reproductive stage promotes panicle branching and higher grain yield in rice (Miura et al., 2010). LAX PANICLE 1 (LAX1) is a grass-specific basic helix–loop–helix (bHLH) transcription factor involved in rice reproduction through the auxin-related pathway. LAX1 interacts with LAX2, a plant-specific nuclear protein, to regulate the formation of axillary meristems during reproduction. The *lax1*, *lax2*, *lax1*/*lax2* single or double mutants dramatically reduce the number of inflorescence branches and no initiation of lateral spikelet (Komatsu et al., 2001; Komatsu K. et al., 2003; Oikawa and Kyozuka, 2009; Tabuchi et al., 2011). The MADS-box containing transcription factors is also associated with inflorescence and panicle branching in rice. The *osmads34* mutants develop abnormal inflorescences and panicles in rice, such as an increased number of primary branches and a decreased number of secondary branches (Zahn et al., 2005; Gao et al., 2010). Suppression of the *OsMADS14*, *OsMADS15*, and *OsMADS18* genes by RNA interference causes a slight delay in reproductive transition. Further depletion of *PAP2* function from these triple knockdown plants inhibited the transition of the meristem to the IM (Kobayashi et al., 2012). OsMADS50 and OsMADS56 (homologous of Suppressor of Overexpression of CONSTANS 1 (SOC1) in *Arabidopsis*) and OsMADS22, OsMADS47, and OsMADS55 (homologous of Short Vegetative Phase (SVP) or AGAMOUS-Like 24 (AGL24) in *Arabidopsis*) are involved in regulating of inflorescence branching by repressing the expression of *Reduced Culm Number 4* (*RCN4*), a rice Terminal Flower 1 (TFL1) homolog (Zhu et al., 2022). Knockdown expression of *OsMADS50*, *OsMADS56*, *OsMADS22*, *OsMADS47*, and *OsMADS55* in *osmads34* mutant results in an increase in inflorescence branching and the number of secondary and tertiary branches (Liu et al., 2013).

The transition from IM to SM is important for panicle architecture establishment in rice. Aberrant Panicle Organization 1 (APO1), an F-box protein, controls spikelet numbers by inhibiting the transition from IM to SM. The *apo1* mutant exhibits decreased number of PBs and spikelets, shortened main axis, and abnormal floral organs (Ikeda et al., 2005; Ikeda et al., 2007). APO1 interacts with APO2 to regulate the development of rice panicles (Abe et al., 2005; Ikeda et al., 2007). The *apo2* inflorescence is similar to the *apo1* inflorescence, as both exhibit reduced numbers of spikelets and abnormal floral organs (Ikeda et al., 2005; Ikeda-Kawakatsu et al., 2012). TAWAWA 1 (TAW1) belongs to a nuclear protein in *Arabidopsis* Light-dependent Short Hypocotyls 1 (LSH1) and *Oryza* Long Sterile Lemma 1 (G1) (ALOG) family. TAW1 regulates rice panicle development by suppressing the transition from IM to SM. TAW1 induces the expression of members of the *SVP* subfamily of MADS-box genes, including *OsMADS22*, *OsMADS47*, and *OsMADS55*. The dominant mutant, *tawawa1-D*, shows prolonged branch formation and increased numbers of spikelets because the activity of the IM is extended, and spikelet specification is delayed. In contrast, reductions in TAW1 activity cause IM abortion and spikelet formation, resulting in the formation of small inflorescences (Yoshida et al., 2013).

AP2 and MADS-box transcription factors are involved in the control of the transition from SM to floral meristems (FMs) in rice. The spikelet is the basic unit of rice panicles. Each spikelet forms one to several small flowers or florets (Yoshida and Nagato, 2011). Rice Indeterminate Spikelet 1 (OsIDS1) is a member of the IDS1 subgroup of the AP2 transcription factors that control inflorescence branching, the initiation of spikelet meristems, and floral meristems (Lee and An, 2012). Rice Supernumerary Bract (SNB) is also a member of the IDS1 subgroup in the AP2 family. Mutations of *SNB* delay the transition from SM to FM and cause the development of multiple rudimentary glumes in an alternative phyllotaxy as well as aberrant floral shape (Lee et al., 2007). The *snb osids1* double mutant generates fewer inflorescence branches and spikelets and delays the transition to FMs (Lee and An, 2012). Frizzy Panicle (FZP)/BRANCHED FLORETLESS 1 (BFL1) is the rice ethylene-response factor (ERF) subfamily of AP2 transcription factors. FZP determines the transition from SM to FM by suppressing the formation of axillary meristems within the spikelet meristem and triggering subsequent floral meristem identity specification. In *fzp* mutant, the formation of florets is replaced by a sequential round of branching (Komatsu M. et al., 2003; Zhu et al., 2003). Multi-Floret Spikelet 1 (MFS1) is also a factor in the grass-specific ERF subfamily of AP2 transcriptional factors. MFS1 plays important roles in regulating the transformation of spikelet meristems to floral meristems by promoting the expression of *SNB*, *OsIDS1*, and *G1*/Elongated Empty Glume (ELE). The *mfs1* mutant shows a delayed transition of spikelet meristems to floral meristems, resulting in an extra hull-like organ and an elongated rachilla (Yoshida et al., 2009; Ren et al., 2013). TONGARI-BOUSHI 1 (TOB1), a protein in the plant-specific YABBY transcription factor family, plays an important role in regulating lateral organ development and spikelet meristem maintenance in rice (Tanaka et al., 2012).

In addition, Rice Circadian Clock Associated 1 (OsCCA1) positively regulates the expression of *Teosinte Branched 1* (*OsTB1*), also known as *Fine Culm 1* (*FC1*), *Dwarf 14* (D14), and *IPA1/OsSPL14* to repress tiller-bud outgrowth. OsCCA1 also regulates IPA1 expression to mediate panicle and grain development (Wang et al., 2020). *Rice Apical Spikelet Abortion* (*OsASA*) encodes a boric acid channel protein that showed the highest expression in inflorescence, peduncle, and anther. The boron distribution maintained by *OsASA* is required for normal panicle development in a process that involves modulating reactive oxygen species (ROS) homeostasis and salicylic acid (SA) biosynthesis (Zhou D. et al., 2021).

Rice panicle development is a complex process. Although many genes related to panicle development have been cloned, its potential molecular mechanism and genetic regulation network are still unclear. Here, we obtained a mutant with abnormal panicles, named *branch one seed 1-1* (*bos1-1*). The *bos1-1* mutant showed pleiotropic panicle defects in panicle development, such as a reduced number of primary and secondary branches and abortion of lateral spikelets, causing a decrease in grain yield. We isolated *BOS1* gene through a positional cloning approach, which encoded a bHLH transcription factor. BOS1 protein was mainly localized in the nucleus. *BOS1* is expressed in young panicles but is expressed at a lower level in other detected tissues and organs and can be induced by phytohormones. BOS1 controlled panicle development by regulating the expression of *OsPIN-FORMED 2* (*PIN2*), *OsPIN3*, *APO1*, and *FZP*. Genomic variation, haplotype, and haplotype network analyses showed that BOS1 had several genomic variations and haplotypes. These results laid the foundation for further exploring the functions of BOS1 in panicle development in rice.

## 2 Materials and methods

### 2.1 Plant materials and growth conditions

A japonica rice variety named Shen Nong 9816 (SN9816), *bos1-1* mutant in SN9816 background, and a Japanese indica rice variety named Habataki were used as materials in the study. SN9816 variety is widely cultivated in northern China and was used as wild type (WT). The *bos1-1* mutant was selected and obtained in the M_2_ segregation population mutated by ethyl methanesulfonate (EMS) (Zhu et al., 2021). Habataki was used as the male parent to generate the F_2_ segregation mapping population. WT, *bos1-1* mutant, Habataki, F_2_ population used for genetic analysis, and F_2_ population used for positional cloning were planted in the experimental station at Shenyang Agricultural University, China (longitude, 123.38°E; latitude, 41.8°N). Rice seeds were first soaked and germinated. Then, the germinated seeds were sown on the seedbed to nurse seedlings. When the seedlings grew to the four-leaf stage, they were transplanted to the paddy field.

To investigate the agronomic traits, WT and *bos1-1* mutant were transplanted in three plots in the paddy field. Each plot contains 20 plants. When the rice materials were in the mature stage, five plants of WT and *bos1-1* mutant from each plot were selected and sampled for the agronomic trait analysis.

### 2.2 Genetic analysis and map-based cloning of *bos1-1* mutation

To study the inheritance of *bos1-1* mutation, we crossed *bos1-1* mutant to WT to generate the first filial generation (F_1_). Then, the F_1_ individuals were self-pollinated, and the second filial generation (F_2_) segregation population was produced. The number of individuals with *bos1-1* phenotype and the total number of plants in the F_2_ population were investigated to calculate the segregate ratio and perform the χ^2^ test.

To find the locus with *bos1-1* mutation, we utilized a positional or map-based cloning strategy. The F_2_ segregation mapping population was generated by crossing *bos1-1* mutant with Habataki. The bulk segregant analysis (BSA) strategy was applied for crude mapping (Yan et al., 2022). The DNA of the F_1_ hybrid (WT × Habataki) and the DNA pool made from 50 plants with *bos1-1* mutant phenotype were used as templates for PCR with molecular markers evenly distributed on the 12 chromosomes of rice. After crude mapping, fine mapping was conducted by developing markers by using polymorphisms between WT and Habataki and enlarging the F_2_ mapping population. Then, the open reading frames (ORFs) in the region *bos1-1* mutation located were amplificated and sequenced to identify *bos1-1* mutation.

### 2.3 MutMap strategy by high-throughput sequencing

To accelerate the mapping processes, the MutMap strategy was also used in the study. Thirty plants with *bos1-1* phenotype in the F_2_ population from *bos1-1* × WT and WT were used for MutMap as described in the previous study (Abe et al., 2012).

### 2.4 Complementation test

For complementation tests, the CDS of *BOS1* fused with a green fluorescent protein (GFP) and the 1,795-bp promoter upstream of ATG were cloned into the pCambia 1300 binary vector with *Xba*I/*Bam*HI and *Pst*I/*Spe*I enzymes, respectively. Then, the *BOS1p*:*BOS1c*-*GFP* plasmid was transformed into a *bos1-1* mutant through the agrobacterium (*Agrobacterium tumefaciens*)-mediated transformation of rice mature embryos (Li et al., 2015; Zhu et al., 2021). In the second and third filial transgenic generations (T_2_ and T_3_), the phenotypes of the complementary transgenic lines harboring *BOS1p*:*BOS1c*-*GFP* construction were observed. Primers used in the plasmid construction are listed in **Supplementary Table S1**.

### 2.5 Gene expression analysis

Gene expression was analyzed by the method described before (Wang et al., 2012; Cui et al., 2017). In brief, total RNA was extracted from 100 mg of SAM, 2-mm panicle, and 3–5-mm panicle using Takara RNAiso Plus (Takara Bio Inc., Otsu, Japan; Cat. No. 9108). Total RNA measuring 20 μg was treated with RNase-free DNase I to remove the DNA contaminated (Promega, Madison, WI, USA; Cat. No. M6101). After treatment, 3 μg of RNA was used for first-strand cDNA synthesis with RevertAid First Strand cDNA Synthesis Kit (Thermo Scientific, Waltham, MA, USA; Cat. No. K1621). *OsACT1* (Os03g0718100) was used as a control. Three biological replicates are performed. The formula, 2^−delta Ct^ was used to calculate the relative expression level of genes. Primers in the list were used for the assay (**Supplementary Table S2**).

Microarray data in Rice Expression Profile Database (RiceXPro, http://ricexpro.dna.affrc.go.jp/) were assayed for the expression of *BOS1*. The expression of *BOS1* in tissues and organs throughout the entire growth period in the field was selected. The expression of *BOS1* in tissues and organs, such as roots, stems, leaf blades, leaf sheath, and inflorescence, were selected for the assay. The expression of *BOS1* in vegetative, reproductive, and ripening stages at 12:00 was also analyzed; three biological replicates were performed. Four-leaf-old seedlings of rice were treated with 10 μM of IAA, 10 μM of gibberellin 3 (GA3), 1 μM of CK, 1 μM of brassinosteroid (BR), 50 μM of abscisic acid (ABA), and 100 μM of jasmonic acid (JA) for 0, 1, 3, 6, and 12 h; two biological replicates were performed. The shoot of the treated seedlings was used for microarray analysis. The signal intensity detected during hybridization was used for drawing heatmap with GraphPad Prism7 software.

### 2.6 Subcellular localization analysis

The *BOS1* CDS fused with GFP driven by 35S promoter was cloned into pCambia 1300, generating *35S:BOS1*-*GFP* construct. The plasmid harboring *35S:BOS1*-*GFP* cassette was transiently expressed in *Nicotiana benthamiana* by infiltration according to the method reported before (Wang et al., 2012). In brief, the constructed *35S:BOS1*-*GFP* binary vector was transformed into *Agrobacterium* strain GV3101 and infiltrates *N. benthamiana* healthy leaves of 4-week-old seedlings. The pCAMBIA1300-P19 plasmid was co-infiltrated to inhibit RNA interference. The infiltrated *N. benthamiana* seedlings were grown in a greenhouse with 16-h light/8-h dark at 26°C. After 3 days, the subcellular localization of BOS1 was observed by laser scanning confocal microscopy.

### 2.7 Haplotype and haplotype network analyses

The genomic DNA variation, haplotype, and haplotype network of *BOS1* were analyzed by using RiceVarMap2 (http://ricevarmap.ncpgr.cn/) website. In the haplotype network analysis of *BOS1*, we selected classification 3, including Indica I, Indica II, Indica III, Indica Intermediate, Aus, Temperate Japonica, Tropical Japonica, Japonica Intermediate, and Intermediate (Intermediate group includes Aromatic and other accessions) for analysis (Huang et al., 2012; Zhao et al., 2015; Zhao et al., 2021).

### 2.8 Statistics analysis

Two-tailed unpaired *t*-test with Welch’s correction was used for statistical analysis. Asterisks indicate significant differences between treatments and controls (**p < 0.01, *p < 0.05). The ns represents no significant differences (*p* > 0.05) between treatments and controls.

### 2.9 Accessions

The gene accessions are listed in **Supplementary Table S3**.

## 3 Results

### 3.1 The panicle development was affected by *bos1-1* mutation

**Figure 1:**
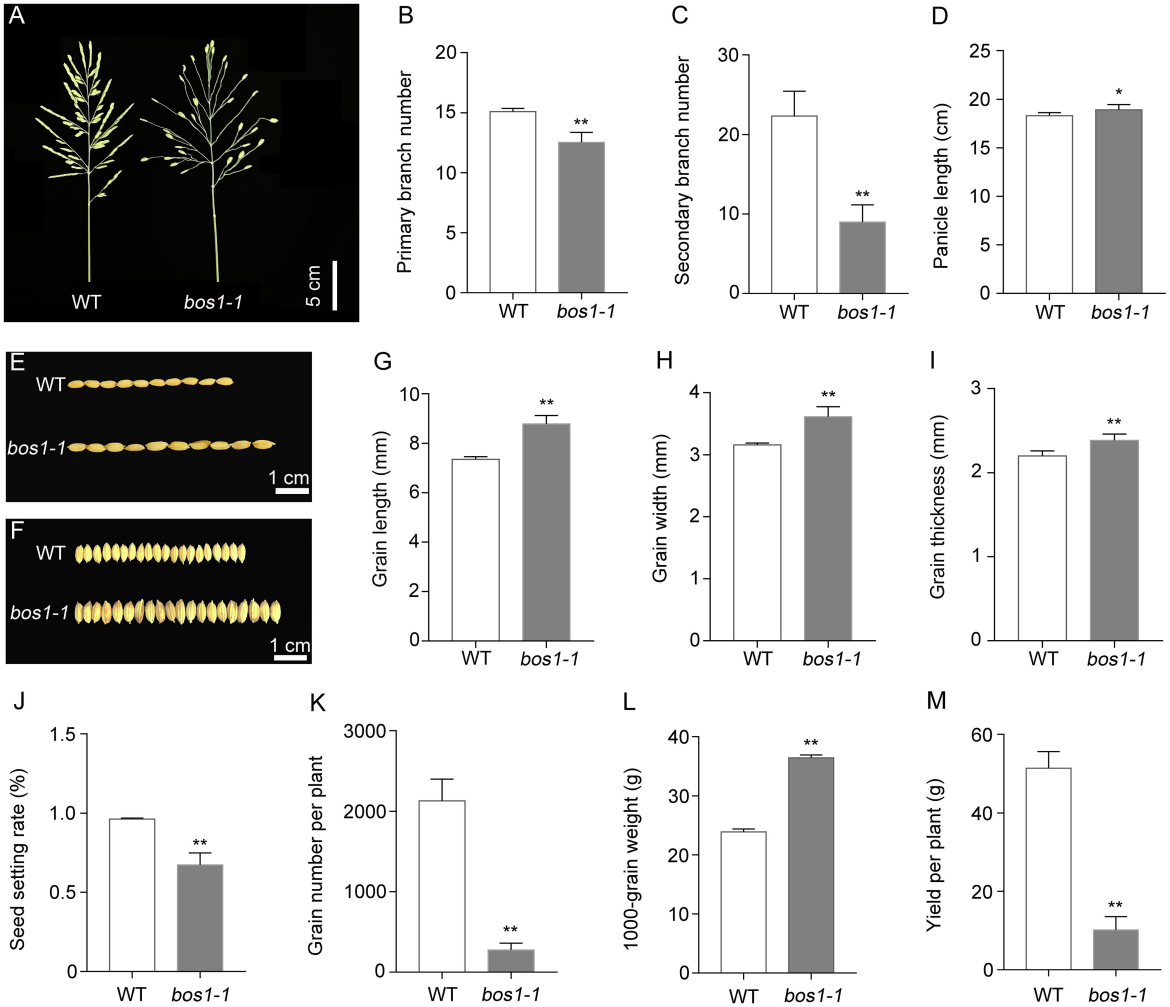
The *bos1-1* mutation confers abnormal panicle phenotype in rice. **(A)** Photos of WT and *bos1-1* panicles. Scale bar is 5 cm. **(B)** Primary branch number. **(C)** Secondary branch number. **(D)** Panicle length. **(E–I)** Grain features include grain length **(E, G)**, grain width **(F, H)**, and grain thickness **(I)**. Scale bar is 1 cm. **(J)** Seed setting rate. **(K)** Grain number per plant. **(L)** 1,000-grain weight. **(M)** Yield per plant. Data in panels **(B–D, G–M)** are means ± s.d. The s.d. represents standard deviation. A two-tailed unpaired *t*-test with Welch’s correction is used for statistical analysis. Asterisks indicate significant differences between WT and *bos1-1* mutant (*p < 0.05, **p < 0.01; Student’s *t*-test). WT, wild type.

A rice panicle consists of a main axis, PBs, SBs, and spikelets. The primary branches are arranged in a spiral phyllotaxy, and spikelets are produced on both the primary and secondary branches (Yoshida et al., 2012). In this study, we identified a mutant with defects in panicle development termed *bos1-1* mutant by using EMS mutagenesis. The mutation of *BOS1* conferred abnormal pleiotropic panicle phenotypes (**Figure 1A**). The main defects in *bos1-1* mutant were that PBs and SBs only had one terminal spikelet and no lateral spikelets (**Figure 1A**). Compared with WT, the number of PBs and SBs were significantly decreased by *bos1-1* mutation, especially the number of SBs (**Figures 1B, C**). The panicle length was increased (**Figure 1D**). Grain sizes, such as grain length, width, and thickness, also increased by *bos1-1* mutation (**Figures 1E–I**). To analyze the effects of *bos1-1* mutation on rice yield, seed setting rate, grain number per plant, and 1,000-grain weight were investigated. Compared with WT, the seed setting rate and grain number per plant of *bos1-1* mutant were significantly reduced, while the 1,000-grain weight was significantly increased (**Figures 1J–L**). Finally, compared with WT, the yield per plant of the *bos1-1* mutant decreased significantly (**Figure 1M**); these results indicate that BOS1 was a positive regulator of rice yield.

### 3.2 Other agronomic traits were also changed in *bos1-1* mutant

**Figure 2:**
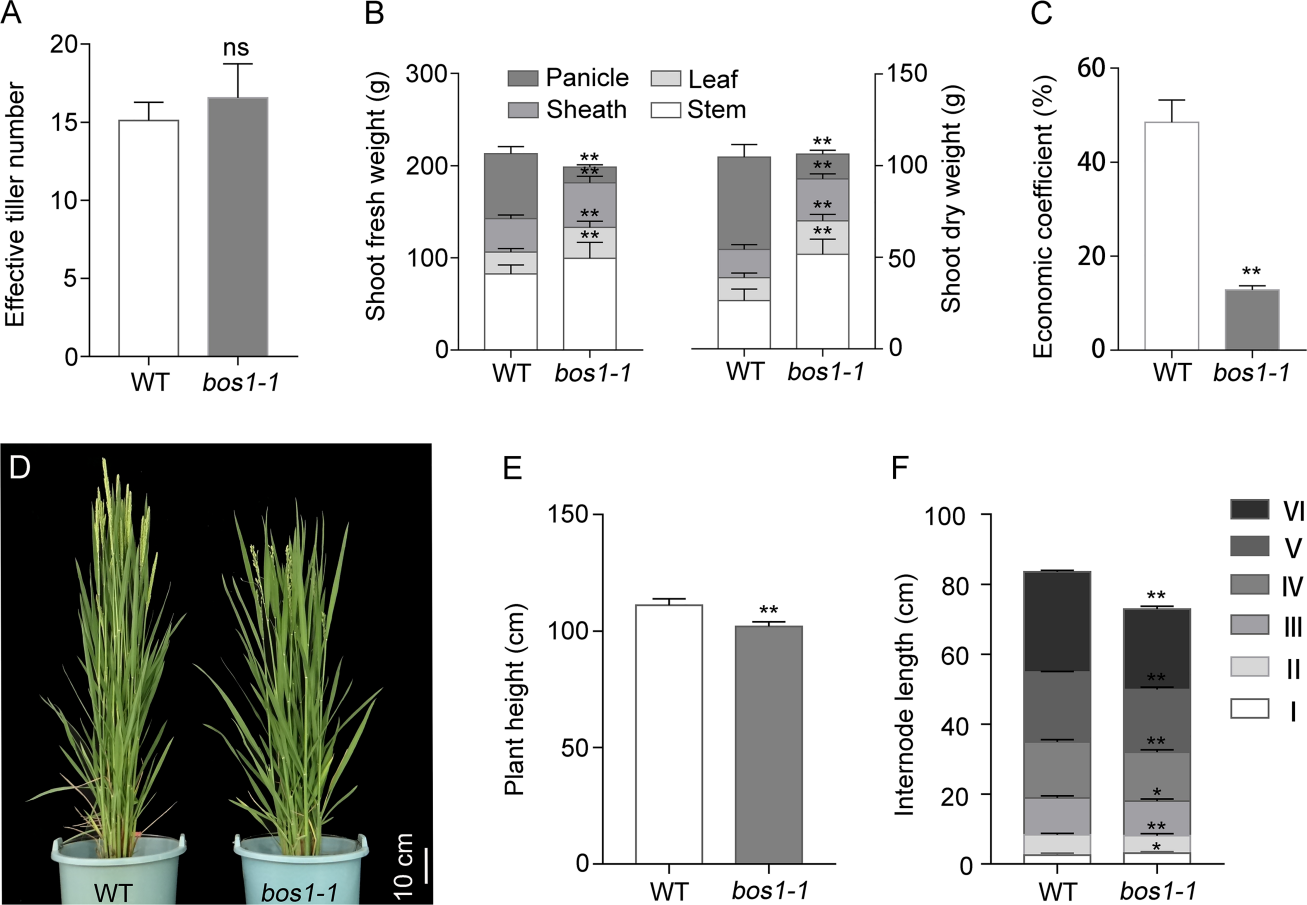
Biomass production in WT and *bos1-1* mutant. **(A)** Effective tiller number. **(B)** Fresh and dry weight/plant. **(C)** Economic coefficient. **(D–F)** Agronomic traits, including photos of rice plant height **(D)**, plant height **(E)**, and internode length **(F)**. Scale bar is 10 cm. Data in panels **(A–C, E, F)** are means ± s.d. The s.d. represents standard deviation. A two-tailed unpaired *t*-test with Welch’s correction is used for statistical analysis. Asterisks indicate significant differences between WT and *bos1-1* mutant (*p < 0.05, **p < 0.01; Student’s *t*-test). ns indicates no significant difference. WT, wild type.

Panicle number per plant is an important factor in rice yield composition, which is determined by the number of effective tillers. Compared with WT, the number of effective tillers of the *bos1-1* mutant was not changed (**Figure 2A**). To determine whether *bos1-1* affects biomass production, the dry weight and fresh weight of different organs above ground were measured. Compared with WT, the dry and fresh weights of stems, leaves, and sheaths of *bos1-1* mutant increased significantly, while the dry and fresh weights of panicles decreased significantly (**Figure 2B**). Therefore, the economic coefficient of *bos1-1* was lower than that of WT (**Figure 2C**). We also investigated the effects of *bos1-1* mutation on plant height and internode length. Compared with WT, the plant height of *bos1-1* mutant was significantly reduced (**Figures 2D, E**), the length of the first elongated internode of *bos1-1* mutant increased significantly, and the length of the second, third, fourth, fifth, and sixth elongated internodes decreased significantly (**Figure 2F**). These results showed that the mutation of *BOS1* significantly reduced the economic yield and shortened plant height by reducing internode length in rice.

### 3.3 The *bos1-1* mutation was mapped through the positional cloning and MutMap strategy

To isolate the locus with *bos1-1* mutation, it is necessary to determine whether the mutation is dominant or recessive and controlled by single or multiple genes. To carry out genetic analysis, we crossed *bos1-1* mutant to WT and generated the first filial (F_1_) hybrid. The phenotype of all the F_1_ individuals was consistent with that of WT. After self-pollination of the F_1_, we obtained an F_2_ segregating population. In the F_2_ population, 830 and 270 progenies exhibited WT and *bos1-1* mutant phenotypes, respectively. The ratio of individuals with WT phenotype to those with *bos1-1* phenotype is 3.07: 1, χ^2^ = 0.1212 < χ^2^ (*P*_0.05, 1_ = 3.84), suggesting that *bos1-1* is a single recessive mutation (**Table 1**).

**Table 1:** Genetic analysis of *bos1-1* mutation.

Cross combination	Plants with WT phenotype	Plants with *bos1-1* phenotype	Total plants	Plants with WT phenotype: plants with *bos1-1* phenotype	χ^2^
*bos1-1 ×* WT	830	270	1,100	3.07:1 ≈ 3:1	0.1212
χ^2^ = 3.84, *p* = 0.05, df = 1		χ^2^ = 6.64, *p* = 0.01, df = 1		χ^2^ < χ^2^ (*p* = 0.05, df = 1)	

WT, wild type.

**Figure 3:**
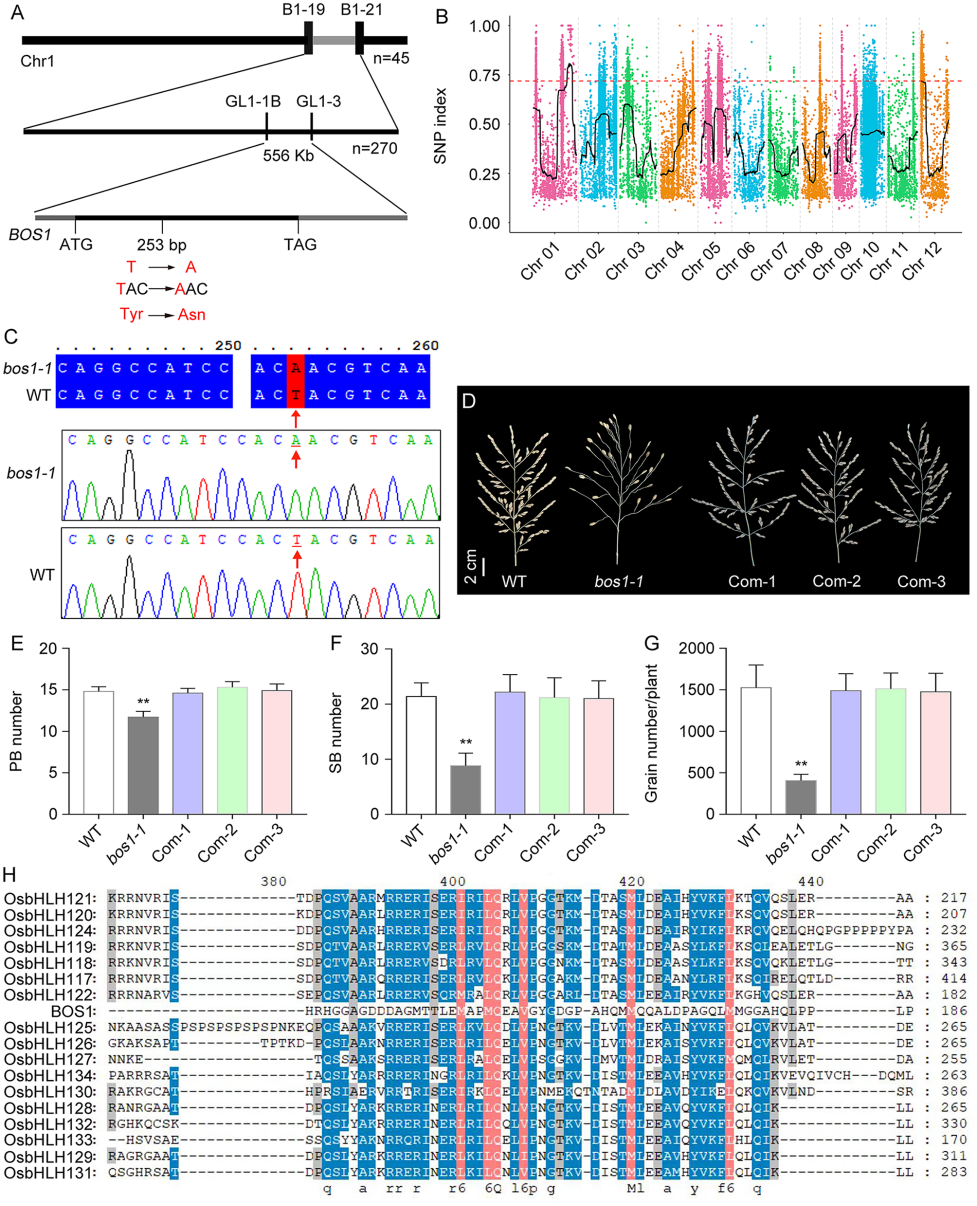
Cloning of *BOS1* gene through positional cloning and MutMap strategies. **(A)** Positional cloning of *BOS1* gene. Upper and middle panels represent crude and fine mapping results of *bos1-1* mutation, respectively. The numbers of plants with *bos1-1* mutant phenotypes used and the molecular markers are shown. Lower panel shows the gene structure of BOS1. Black line represents exons. Gray lines represent the 5′ untranslated region (5′UTR) and 3′UTR. **(B)** SNP index obtained by MutMap through high-throughput sequencing. **(C)** Sequence alignment and the trace file showing the mutation identified by sequencing in WT and *bos1-1*. **(D)** Photos of panicles in WT, *bos1-1*, and three complementary transgenic lines. Scale bar is 1 cm. **(E, F)** The number of PBs **(E)**, SBs **(F)**, and grains **(G)**. **(H)** Sequence alignment of BOS1 protein and other 17 closely homologous bHLH proteins. SNP, single-nucleotide polymorphism; WT, wild type; PBs, primary branch; SBs, secondary branches. Data in E-G are means ± s.d.. The s.d. represents standard deviation. A two-tailed unpaired t-test with Welch’s correction is used for statistical analysis. Asterisks indicate significant differences (**P < 0.01; Student’s *t*-test).

To isolate *bos1-1* mutation, we applied the positional cloning method. We crossed *bos1-1* mutant to an indica rice variety Habataki and produced F_1_. After the self-pollination of F_1_, the F_2_ segregation population was created. BSA was used to map bos*1-1* mutation crudely. In the F_2_ population, 45 individuals with the *bos1-1* mutant phenotype were selected to create a DNA pool. Both the F^1^ (a hybrid of WT × Habataki) DNA and the DNA pool were used as a template to PCR with the polymorphic markers evenly distributed in the 12 chromosomes of rice. The *bos1-1* mutation was primarily located between B1-19 and B1-21 molecular markers on chromosome 1, with a physical distance of 4.35 Mb. Subsequently, 270 individual plants with *bos1-1* mutant phenotypes were used for fine mapping. The *bos1-1* mutation was located between GL1-1B (Chr. 01: 35,424,703) and GL1-3 (Chr. 01: 35,981,090) molecular markers, with a physical distance of 556 kb (**Figure 3A**).

In the study, we also used the MutMap strategy, a method based on whole-genome resequencing of pooled DNA from a segregating population of plants with mutant phenotypes, to accelerate gene cloning (Abe et al., 2012). Thirty plants with *bos1-1* mutant phenotypes in F^2^ genetic analysis population (*bos1-1* × WT) were chosen to isolate DNA and made a pool to conduct high-throughput sequencing together with WT using an Illumina GAIIx sequencer.

Approximately 54.56 and 84.89 million raw reads, 54.56 and 84.84 million clean reads with an average length of 150 bp, 109.05 and 169.68 million total reads, and 16.34 and 25.42 Giga clean bases with a Q30 > 93% and 41% GC content were obtained from WT and the pool of *bos1-1* mutant, respectively. Approximately 99% and 96% of the total reads were mapped and properly mapped to the rice reference genome (IRGSP-1.0, https://rapdb.dna.affrc.go.jp/download/irgsp1.html) (**Supplementary Table S4**). The average depth (coverage) of the high-throughput sequencing was 41 and 65 for WT and the pool of *bos1-1* mutant, respectively. The cover ratio of the high-throughput sequencing was 92% to 97% for WT and the pool of *bos1-1* mutant (**Supplementary Table S5**).

Single-nucleotide polymorphisms (SNPs) and insertion–deletions (indels) between WT and the pool of *bos1-1* mutants were analyzed and annotated (**Supplementary Figure S1**). The association of the SNPs with the *bos1-1* mutation was performed by using the SNP index, which was the ratio between the number of reads of a mutant SNP and the total number of reads corresponding to the SNP (Abe et al., 2012). One region was linked with the *bos1-1* mutation located in chromosome 1 (Chr. 1) with 4.83 Mb (**Figure 3B**, **Supplementary Table S6**).

Approximately 280 differential SNPs were located in Chr. 1. The Chr. 1 region was co-located with the locus mapped by the positional cloning method, suggesting that *bos1-1* mutation was in Chr. 1 between GL1-1B and GL1-3 markers. In the co-located region, 30 differential SNPs were identified by the MutMap method in the pool with the *bos1-1* mutant phenotype, among which three SNPs led to non-synonymous mutations in three genes, including Os01g0828300, Os01g0831000, and Os01g0836600. Then, the coding regions of the three candidate genes were amplified and sequenced. A T-to-A mutation at 253 bp downstream of ATG was identified, which changed the codon from TAC to AAC, resulting in the change of the 85th amino acid from tyrosine (Tyr) to asparagine (Asn) (**Figures 3A, C**).

### 3.4 Genomic DNA of *BOS1* was able to rescue the defects of *bos1-1* mutant

To confirm that the mutation in *BOS1* (Os01g0831000) locus was *bos1-1*, a plasmid containing the *BOS1* CDS fused to GFP driven by the native promoter of *BOS1* genes (*BOS1_P_:BOS1_C_-GFP*) was constructed. The resulting plasmid was transformed into *bos1-1* mutant to obtain the complementary transgenic lines. The *bos1-1* mutant phenotypes, such as the number of PBs, SBs, and grains, were rescued in the complementary transgenic lines, indicating that the mutation in *BOS1* is responsible for the *bos1-1* mutant phenotype (**Figures 3D–G**). *BOS1* encodes a known protein, LAX1, which is a grass-specific basic bHLH domain-containing transcription factor (Komatsu et al., 2001; Komatsu K. et al., 2003; Oikawa and Kyozuka, 2009). A point missense mutation in the bHLH domain disrupted the functions of BOS1.

The rice genome contains 167 bHLH domain-containing proteins, which can be divided into 22 (A to V) subfamilies (Li et al., 2006). bHLH proteins in A and B subfamilies including BOS1 (OsbHLH123) were selected to conduct protein sequence alignment. The results showed that the bHLH proteins were highly conserved, while BOS1 showed lower conservation with other members, even in the bHLH domain (**Figure 3H**).

### 3.5 *BOS1* encoded a nuclear protein, and its expression was induced by hormones

**Figure 4:**
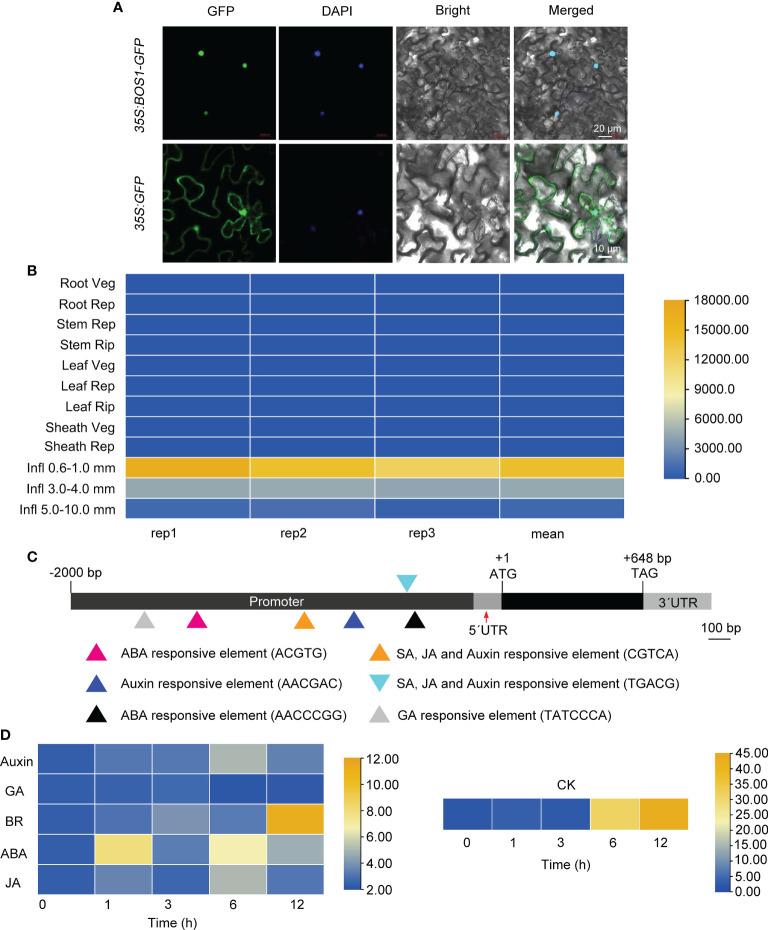
Subcellular localization of BOS1 protein and analysis of *BOS1* gene expression profiles. **(A)** Representative microscopic images showing the subcellular localization of green fluorescent protein (GFP) and BOS1-GFP fusion protein transiently expressed in *Nicotiana benthamiana* leaves driven by 35S promoter. DAPI was used as a nuclear marker. Scale bars are 20 and 10 μm as labeled. **(B)** Spatial–temporal of *BOS1* gene expression of various tissues/organs throughout entire growth in the field. Three biological replications were performed. **(C)** Hormone response elements in the promoter region of *BOS1*. Triangles in different colors represent the elements identified. **(D)** Heat map of *BOS1* gene expression pattern under auxin, GA, CK, BR, ABA, and JA treatment. Two biological replications were performed. CK, cytokinin; BR, brassinosteroid; ABA, abscisic acid; JA, jasmonic acid.

To determine the subcellular localization of BOS1, a binary plasmid containing the full-length CDS of *BOS1* fused to GFP driven by Cauliflower Mosaic Virus (CaMV) 35S constitutive promoter, *35S:BOS1*-*GFP*, was created. The constructed *35S:BOS1*-*GFP* binary plasmid was transferred into an agrobacterium (*A. tumefaciens*) strain GV3101 and infiltrated healthy *N. benthamiana* seedling leaves for transient expression. The results showed that BOS1 was mainly localized in the nucleus, indicating that BOS1 is a nuclear localization protein (**Figure 4A**).

Previous studies have shown that the *BOS1* gene is expressed at the boundary between SAM and the new meristem region, indicating that *BOS1* gene is a major regulator of axillary meristem formation (Komatsu et al., 2003; Matin and Kang, 2011). To analyze the expression of *BOS1* gene, we used the microarray data from the RiceXpro database. The expression of *BOS1* in inflorescence was higher than that in other tissues and organs, which corresponds to the severe inflorescence phenotype of the *bos1-1* mutant (**Figure 4B**). To understand whether BOS1 is induced by hormones, we also analyzed *cis*-acting elements in the *BOS1* promoter. The *cis*-acting elements’ response to hormones was identified (**Figure 4C**). Then, the expression of *BOS1* gene under different hormone treatments, including IAA, GA3, CK, BR, ABA, and JA, was examined. The results showed that the expression of *BOS1* was induced by hormones, especially by auxin, CK, BR, ABA, and JA (**Figure 4D**).

### 3.6 The *bos1-1* mutation changed the expression of genes related to inflorescence development

**Figure 5:**
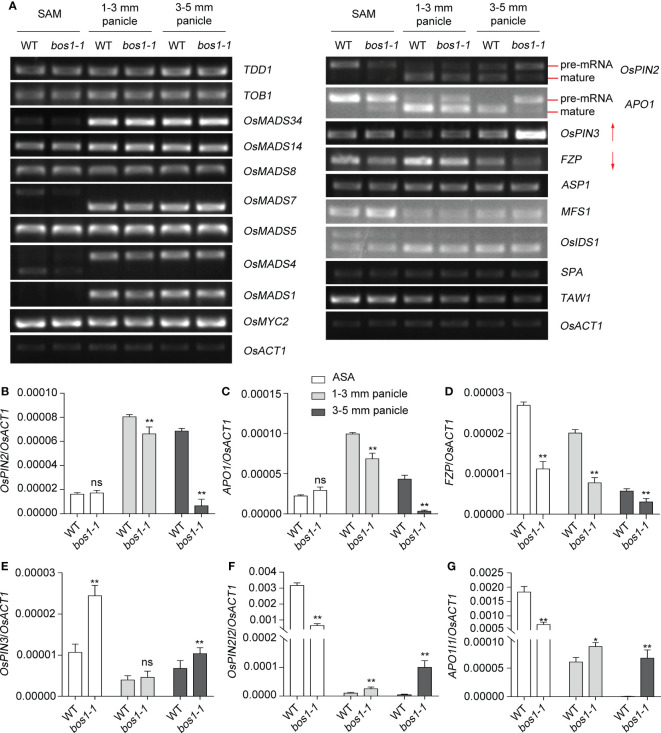
Expression of genes related to panicle development in WT and *bos1-1* mutant. **(A)** The expression of genes related to panicle development in WT and *bos1-1* mutant by using semi-RT-PCR. Around the shoot apex (ASA), 1–3-mm panicles, and 3–5-mm panicles were sampled for the assay. The pink arrow indicates that the alternative splicing of FZP and APO1 genes has changed. **(B–E)** The mature transcripts of genes *OsPIN2* **(B)**, *APO1* **(C)**, *FZP* **(D)**, and *OsPIN3* **(E)** detected by quantitative RT-PCR. **(F, G)** The intron retention transcripts of genes *OsPIN2I2* **(F)** and *APO1I1* **(G)** detected by quantitative RT-PCR. Three biological replicates were performed. *OsACT1* (Os03g0718100) was employed as an endogenous control. Data in panels B–G are means ± s.d. The s.d. represents standard deviation. A two-tailed unpaired *t*-test with Welch’s correction is used for statistical analysis. Asterisks indicate significant differences between WT and *bos1-1* mutant (*p < 0.05, **p < 0.01; Student’s *t*-test). ns indicates no significant difference. Primers used in the assay are listed in **Supplementary Table S2**.

To explore the molecular mechanisms whereby BOS1 controls panicle development in rice, the expression of 19 genes associated with inflorescence development was examined by reverse transcription–polymerase chain reaction (RT-PCR). The results show that the expression of 15 genes in the *bos1-1* mutant was similar to that in WT; however, the expression of *OsPIN2*, *OsPIN3*, *APO1*, and *FZP* genes was changed by *bos1-1* mutation (**Figure 5A**). The alternative splicing of *OsPIN2* and *APO1* was altered in *bos1-1* mutant, leading to increasing pre-mRNA and reducing mature transcripts of them (**Figures 5B–E**). The expression of *OsPIN3* gene was upregulated in *bos1-1* mutant (**Figure 5F**); however, the expression of *FZP* gene was downregulated in *bos1-1* mutant (**Figure 5G**). The results of their expression patterns showed that higher expression of *OsPIN3* and *FZP* was also in the panicle. The expression of *OsPIN2* in the root was higher than in other organs (**Supplementary Figure S2**). *APO1* was also expressed in SAM and young panicles as reported previously (Ikeda et al., 2005). These results indicated that the expression pattern of *BOS1* was similar to that of *OsPIN3*, *APO1*, and *FZP*.

### 3.7 *BOS1* genomic variation, haplotype, and haplotype network analyses

**Figure 6:**
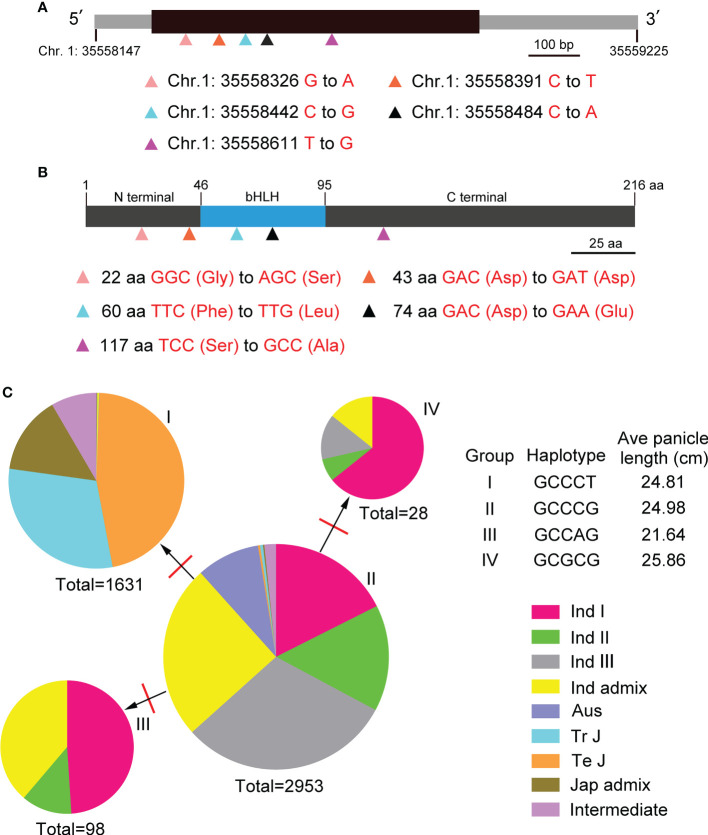
Genetic variation and haplotype network analyses of *BOS1*. **(A)** The gene structure of *BOS1* and location of the five genomic variations. Black box represents exon, and gray boxes represent the 5′ untranslated region (5′UTR) and 3′UTR. Scale bar is 100 bp. **(B)** The protein structure of BOS1 and location of the five genomic variations. Gray boxes represent the N-terminal and C-terminal, and blue boxes represent the bHLH domain. Scale bar is 25 aa. **(C)** A haplotype network of the five genomic variations. The red lines represent the number of mutations between two haplotypes. WT, wild type.

To study genomic variations (GVs) and haplotypes, genomic sequences of *BOS1* gene were analyzed through the RiceVarMap2 (ricevarmap.ncpgr.cn) website. In *BOS1* locus, there were eight genomic variations caused by SNP. Five of the genomic variations were distributed in the exon of *BOS1* gene, four of which were missense mutations (**Figures 6A, B**).

The haplotypes of *BOS1* in 4,726 rice landraces, including Indica I, Indica II, Indica III, Indica Intermediate, Aus, Temperate Japonica, Tropical Japonica, Japonica Intermediate, and Intermediate ecotypes, were also analyzed. Four haplotypes were obtained, and a haplotype network was built (**Figure 6C**, **Supplementary Table S4**). The proportion of haplotype II in the total number of rice ectypes was the largest, which contained all types of rice, indicating that haplotype II was the main haplotype of *BOS1*. One mutation changed haplotype II to haplotype I. Haplotype I had the japonica rice. One mutation changed haplotype II to haplotype III and IV. Haplotypes III and IV only had indica rice ecotypes. In addition, we also found that Aus, Temperature Japonica, and Tropical Japonica only contained two haplotypes, while Indica I and Indica II contained four haplotypes, indicating that *BOS1* has evolved into high diversity in different varieties (**Figure 6C**, **Supplementary Table S7**). We analyzed the average panicle length under different haplotypes and found that the average panicle length of haplotype IV was the longest, the average panicle length of haplotype III was the shortest, and the panicle length of haplotype I and II was similar.

## 4 Discussion

Rice generates raceme panicles and normally comprises primary and secondary branches, which bear single-flowered spikelets (Kyozuka et al., 2014; Zhu et al., 2022). Rice panicle development plays critical roles in yield formation, which is defined by IMs, PBMs, SBMs, SMs, and FMs produced by SAMs and AMs (Itoh et al., 2005; Kyozuka et al., 2014). To further understand the mechanisms of panicle development, a mutant with abnormal panicles, named *bos1-1*, was obtained using EMS mutagenesis in this study. *BOS1* gene was cloned. The regulatory roles of BOS1 on rice panicle development were explored.

### 4.1 A missense mutation in *BOS1* gene disrupted its function

We isolated *BOS1* gene by applying the positional cloning method and MutMap strategy (**Figure 3**). *BOS1* is a small gene, and the CDS of *BOS1* gene was only 648 bp without intron encoding a bHLH domain-containing transcription factor (**Figure 3**). A single amino acid substitution, Tyr to Asn, in the bHLH domain altered BOS1 functions. The *bos1-1* mutation conferred abnormal panicle phenotypes, including abortion of lateral spikelets, increased panicle length and grain size, reduced number of primary branches and secondary branches, and decreased grain number per panicle and seed setting rate (**Figures 1**, **2**). Genomic DNA of *BOS1* was able to recover the defects in *bos1-1* mutant, suggesting mutation in *BOS1* locus is responsible for *bos1-1* mutant phenotype. In rice, *BOS1* was previously cloned and known as *LAX1* (Komatsu et al., 2001; Komatsu K. et al., 2003; Oikawa and Kyozuka, 2009). *BOS1* encodes a bHLH domain-containing transcription factor in the B subfamily (Li et al., 2006). However, compared with other members in the A and B subfamily, the sequence similarity of BOS1 was lower, indicating BOS1 may function independently with other members in the B subfamily (**Figure 3H**). Mutations of *BOS1*/*LAX1* exhibited more severe phenotypes, suggesting that BOS1 plays important roles in panicle development.

### 4.2 *BOS1* expressed in young panicles and encoded a nuclear protein

Previous studies have shown that the *BOS1* gene is expressed at the boundary between SAM and the new meristem region, indicating that *BOS1* gene is a major regulator of axillary meristem formation (Komatsu M. et al., 2003; Matin and Kang, 2011). In this study, we found that *BOS1* was predominantly expressed in young panicles but was expressed at a lower level in other tissues or organs detected, such as root, leaf, sheath, and stem (**Figure 4B**). The *BOS1* expression profile corresponds to the severe panicle phenotypes of *bos1-1* mutant. The vegetative branching of *bos1-1* and *lax1* mutants is normal, but the axillary meristems were severely blocked in the mutants’ reproductive stage, revealing that BOS1 is active in the reproductive stage. The results of subcellular localization showed that BOS1 protein was mainly localized in the nucleus, which is consistent with its potential functions as a transcription factor (**Figure 4A**). In addition, several hormone response elements, including auxin, GA, ABA, SA, and JA response elements, in the *BOS1* promoter were identified, suggesting that *BOS1* may be induced by phytohormones (**Figure 4C**). As expected, the expression of *BOS1* was activated by auxin, BR, ABA, SA, and JA, suggesting that BOS1 may control panicle development by regulating hormone biosynthesis or signaling (**Figure 4D**). More efforts will be put to dissect the principles.

### 4.3 BOS1-regulated panicle development-related gene expression

The bHLH domain-containing proteins are one of the largest transcription factor families having 167 members in rice (Li et al., 2006). The bHLH domain has two adjacent regions with distinctive functions: the basic region and the HLH region. The HLH region mediates protein interaction, allowing the formation of homodimers or heterodimers (Pires and Dolan, 2010). The basic region functions as a DNA-binding motif. It has been known that bHLH domain-containing proteins can bind to *cis*-elements known as E-box, G-box, PIF-binding E (PBE) box, and N-box (Pires and Dolan, 2010). As a potential transcription factor, BOS1 may control rice panicle development by regulating gene expression. In this study, the differential expression of rice panicle development-related genes between WT and *bos1-1* was examined. The results showed that *APO1* and *FZP* were differential expressed. *FZP* determines the transition from SM to FM (Komatsu M. et al., 2003; Zhu et al., 2003). APO1 is a known regulator of the transition from IM to SM (Ikeda et al., 2005; Ikeda et al., 2007; Ikeda-Kawakatsu et al., 2012).

We also found that the expression of *OsPIN2* and *OsPIN3* is differential between WT and *bos1-1* mutant. *OsPIN2* gene encodes an auxin efflux carrier that possibly regulates the auxin from the root tip to the root-elongation zone in rice (Inahashi et al., 2018). Overexpression of *OsPIN2* leads to a shorter plant height, more tillers, and a large tiller angle (Chen et al., 2012). Expression of *OsPIN3* gene is involved in polar auxin transport and induced by osmotic stress in rice. Overexpression of *OsPIN3* improves drought tolerance (Zhang et al., 2012). It is also reported that *OsPIN1* and *OsPIN3* are involved in vegetative axillary meristem specification and outgrowth in rice (Deshpande et al., 2015). Barren Stalk 1 (BA1) is the homolog of BOS1 in maize. BA1 physically interacts with Barren Inflorescence 2 (BIF2) and controls auxin-mediated axillary meristem initiation (Gallavotti et al., 2004; Skirpan et al., 2008). The phenotypes of *ba1* are similar to the *bif2* mutant (Mcsteen and Hake, 2001; Ritter et al., 2002; Skirpan et al., 2008). The expression of *BA1* is induced by auxin. In *ba1* mutant, no auxin accumulation in the newly initiated inflorescence meristems is examined (Wu and Mcsteen, 2007; Gallavotti et al., 2008). These results suggest that *OsPIN2*, *OsPIN3*, *APO1*, and *FZP* may be the direct or indirect targets for BOS1 to control panicle development. More efforts are required to confirm this hypothesis in the future.

### 4.4 *BOS1* locus encountered genomic variations

*BOS1* is a small gene without introns, and the genomic DNA of *BOS1* gene is only 1,078 bp. The results of genomic variation analysis in 4,726 rice accessions showed that eight SNPs were found. Five GVs were distributed in exons, four of which were missense mutations, and the remaining one is synonymous mutations (**Figures 5A, B**). Four haplotypes of the GVs in *BOS1* locus were obtained. Haplotype II was the ancestor one because all types of rice had this haplotype and the frequencies were the highest. The haplotype network of *BOS1* gene indicated that one mutation changed haplotype II to haplotypes I, III, and IV. BOS1 protein sequence was limitedly conserved with other members, even in the bHLH domain (Li et al., 2006). It was interesting that BOS1 contains a divergent non-synonymous polymorphism (Chr. 1: 35558611 T to G). According to the database of RiceVarMap2, 99.70% of indica varieties contain G, whereas 98.20% of japonica varieties contain T. It is likely that BOS1 might be involved in rice subspecies divergence.

In conclusion, panicle defective mutant *bos1-1* was obtained by EMS mutagenesis. Mutations in *BOS1* gene caused severe defects in panicle development and other related agronomic traits. *BOS1* gene was isolated by combining positional cloning and MutMap methods, which encoded a bHLH domain-containing protein. A single amino acid change disrupted the bHLH domain and conferred the *bos1-1* mutant phenotype*. BOS1* gene was predominantly expressed in young panicles and was induced by phytohormones. BOS1 protein was localized in the nucleus. The expression of panicle development-related genes, such as *OsPIN2*, *OsPIN3*, *APO1*, and *FZP*, was changed by *bos1-1* mutation, suggesting the genes may be the direct or indirect targets of BOS1 to regulate panicle development. *BOS1* gene encountered genomic variations. These results laid the foundation to further dissect the functions of BOS1.

## Data availability statement

The datasets presented in this study can be found in online repositories. The names of the repository/repositories and accession number(s) can be found in the article/**Supplementary Material**.

## Author contributions

XW and YL designed the study. YL, XZ and YH generated the genetic and mapping population. YL and XZ prepared the samples for MutMap. YL cloned the gene. YL and SL made the construct and generated the transgenic lines. YL observed the phenotype of the mutant and complementary transgenic lines. YL and YY performed expression pattern and subcellular localization experiments. YL and SL aligned the protein sequence. YL examined the expression of panicle development-related genes by quantitative RT-PCR. YL and YH analyzed the GVs, haplotypes, and haplotype networks. XW and YL prepared the figures and wrote the manuscript. All authors contributed to the article and approved the submitted version.
