# Supplementary material for: BOS1 is a basic helix–loop–helix transcription factor involved in regulating panicle development in rice
Source: Front Plant Sci. 2023 Apr 26;14:1162828. doi: 10.3389/fpls.2023.1162828 (PMC10169713; doi:10.3389/fpls.2023.1162828)
Supplement: Supplementary file 1 [file DataSheet_1.pdf]

Supplementary Materials

BOS1 is a basic helix-loop-helix transcription factor involved in regulating panicle development in rice

Yanpeng Lv, Xinfeng Zhang, Yanjuan Hu, Shuang Liu, Yanbin Yin, Xiaoxue Wang*

*** Correspondence:** Corresponding Author: wangxx@syau.edu.cn or xiaoxuewang6@163.com

1 Supplementary Tables

Supplementary Table S1. Primers used in the binary vector construction

Name	Sequences	Usage
BOS1cF	TCTAGAATGCATGACCCACGCGGC	BOS1 CDS
BOS1cR	GGATCCATAAGATCCTTGCGCACCAG	
BOS1pF	CTGCAGTAAAAAGGGATTGTACGGCTTC	BOS1 promoter
BOS1pF	ACTAGTGTTTTAGTCGTGGTATGGATCCA	

Note: The red Characters represent enzymes used for the vector construction. TCTAGA: XbaI; GGATCC: BamHI; CTGCAG: PstI; ACTAGT: SpeI.

Supplementary Table S2. Primers used in the gene expression assay

Name	Sequences	Usage
BOS1ReF	GCCCTTGAGCTGAACCATC	BOS1, RT-PCR
BOS1ReR	GGTCAGTCTCCTGGACGAAG	
ASP1ReF	GAAGTACCTCAGCGGGTTCA	ASP1, RT-PCR
ASP1ReR	CAACGTCAGCAGCTGTGTTAT	
TDD1ReF	TGATCGAGCAGGAGACCTTC	TDD1, RT-PCR
TDD1ReR	AGCTCCTCGATGAACCTCAC	
TOB1ReF	TCTCCTCTCCCCCACCTC	TOB1, RT-PCR
TOB1ReR	CGCTGGGTAGCCTTGTTG	
OsMADS14ReF	AATGTCAAAAGCACCTCATG	OSMADS14, RT-PCR
OsMADS14ReR	TCTGGACTTTCTGCTTCTCCA	
OsMADS34ReF	AGCTCCACTGGCTACAAATGA	OSMADS34, RT-PCR

OsMADS34ReR	CATTGCTGCAGTTTCCGTTC	
FZPReF	CTCGTACGGTCACCACCAC	FZP, RT-PCR
FZPReR	CTCAGGTACCCGGAGTTGTC	
APO1ReF	CTTCGCCGTCAAGAACATCT	APO1, RT-PCR
APO1ReR	CCTTGCTCCATACGTTCTCC	
MFS1ReF	CGCTTCTCTACAAGGCCATC	MFS1, RT-PCR
MFS1ReR	CTCCTCCAGCGACAGGTTC	
OsIDS1ReF	GGAGGCTGACATCAACTTCAA	OsIDS1, RT-PCR
OsIDS1ReR	CAAGAAGTTGGCCCATGC	
TAW1ReF	CTGGAGTTCCTCCGCTACCT	TAW1, RT-PCR
TAW1ReR	GCCGAAGGGGTTGTTCTC	
OsMADS8ReF	CAACCAGTTGCATGGACAAG	OsMADS8, RT-PCR
OsMADS8ReR	GCAGTCACGCATGAGTTGTT	
OsMADS7ReF	CTGGAGGAAAGCAACCATGT	OsMADS7, RT-PCR
OsMADS7ReR	GTGTTCATGCACGCACTGTT	
OsMADS5ReF	CGAGCAACTTGAGAACCAGA	OsMADS5, RT-PCR
OsMADS5ReR	GGTCACAAATTGCATGATGG	
OsMADS4ReF	GATCGAGAACTCGACGAACC	OsMADS4, RT-PCR
OsMADS4ReR	CTTGTGCTTCTCATCCCAGAG	
OsMADS1ReF	TATGTCCTGGCAAGATGGTG	OsMADS1, RT-PCR
OsMADS1ReR	TGTGTTCATTGGGGTGATGA	
OsIDS1ReF	GGAGGCTGACATCAACTTCAA	OsIDS1, RT-PCR
OsIDS1ReR	CAAGAAGTTGGCCCATGC	
OsMYC2ReF	CCCATGGACATGAAGGACTC	OsMYC2, RT-PCR
OsMYC2ReR	CGAAGTCGGAGAAGTTGAGC	
OsPIN2ReF	ACACATGTTCGTGTGGAGCTC	OsPIN2, RT-PCR
OsPIN2ReR	CCACTCACCCCTGTGGCA	
OsPIN3AReF	CATCACGCTCTTCTCCCTCT	OsPIN3A, RT-PCR
OsPIN3AReR	CGACGAGCTAAGCGAGTAGA	
SPAReF	CTGCTGCTGGTGTTTCAAAG	SPA, RT-PCR
SPAReR	AACTGCGTGAATCCTTGACC	

Supplementary Table S3. Gene accessions

Gene name	ID in RAP-DB	ID in MSU
BOS1	Os01g0831000	LOC_Os01g61480
TDD1	Os04g0463500	LOC_Os04g38950
TOB1	Os04g0536300	LOC_Os04g45330
OsMADS34	Os03g0753100	LOC_Os03g54170
OsMADS14	Os03g0752800	LOC_Os03g54160
OsMADS8	Os09g0507200	LOC_Os09g32948
OsMADS7	Os08g0531700	LOC_Os08g41950
OsMADS5,	Os06g0162800	LOC_Os06g06750
OsMADS4	Os05g0423400	LOC_Os05g34940
OsMADS1	Os03g0215400	LOC_Os03g11614
OsMYC2	Os10g0575000	LOC_Os10g42430
OsPIN2	Os06g0660200	LOC_Os06g44970
OsPIN3	Os01g0643300	LOC_Os01g45550
APO1	Os06g0665400	LOC_Os06g45460
FZP	Os07g0669500	LOC_Os07g47330
ASP1	Os08g0162100	LOC_Os08g06480
MFS1	Os05g0417600	LOC_Os05g34500
OsIDS1	Os03g0818800	LOC_Os03g60430
SPA	Os05g0571000	LOC_Os05g49590
TAW1	Os10g0478000	LOC_Os10g33780
OsACT1	Os03g0718100	LOC_Os03g50885

Supplementary Table S4. Quality analysis of high-throughput sequencing

Sample	Raw reads	Clean reads	Clean bases	Mapped (%)	Properly mapped (%)	Q30 (%)	GC (%)
WT	54,555,342	54,526,746	16,336,315,796	99.03	96.95	93.74	41.68
Pooling DNA*	84,891,025	84,841,406	25,415,276,790	99.12	96.9	93.81	41.47

Note: *The pool DNA was made from 50 plants in F_2 genetic analysis population with *bos1-1* mutant phenotypes.

Supplementary Table S5. Depth and cover ratio analysis of the high-throughput sequencing

Genotype	Average depth	Cover ratio 1× (%)	Cover ratio 5× (%)	Cover ratio 10× (%)
WT	41	96.66	94.66	92.88
Pooling DNA*	65	97.1	95.42	94.08

Note: *The pool DNA was made from 50 plants in F_2 genetic analysis population with *bos1-1* mutant phenotypes.

Supplementary Table S6. SNP associated regions with *bos1-1* mutation

Chromosome ID	Start	End	Size (Mb)	Gene Number
Chr. 1	34,374,575	39,201,562	4.83	887

Supplementary Table S7. Haplotypes of *BOS1* and their frequencies

Group	Haplotype	Total		Indica						Aus		Japonica					Intermediate	
		Accession	Rate (%)	Accession					Rate (%)	Accession	Rate (%)	Accession				Rate (%)	Accession	Rate (%)
				Ind I	Ind II	Ind III	Ind admix	Total				Te J	Tr J	Jap admix	Total			
I	GCCCT	1631	34.51	1	1	1	5	8	0.49	1	0.06	758	492	235	1485	91.05	137	8.40
II	GCCCG	2953	62.48	519	450	902	738	2609	88.35	268	9.08	9	12	6	27	0.91	49	1.66
III	GCCAG	98	2.07	48	12	0	38	98	100	0	0.00	0	0	0	0	0.00	0	0.00
IV	GCGCG	28	0.59	18	2	4	4	28	100	0	0.00	0	0	0	0	0.00	0	0.00

Note: 1. About 4726 rice accessions in the data base. 2. Rate of total accessions of each haplotype is to 4726 accessions in the database. 4. Rate of Indica, Aus, Japonica, and Intermediate accessions is to the total accessions of each haplotype. 3. Ind I, Ind II, Ind III, Ind admix, Te J, Tr J, and Jap admix represent indica I, indica II, indica III, indica intermediate, temperate japonica, tropical japonica, and japonica intermediate, respectively.

2 Supplementary Figures

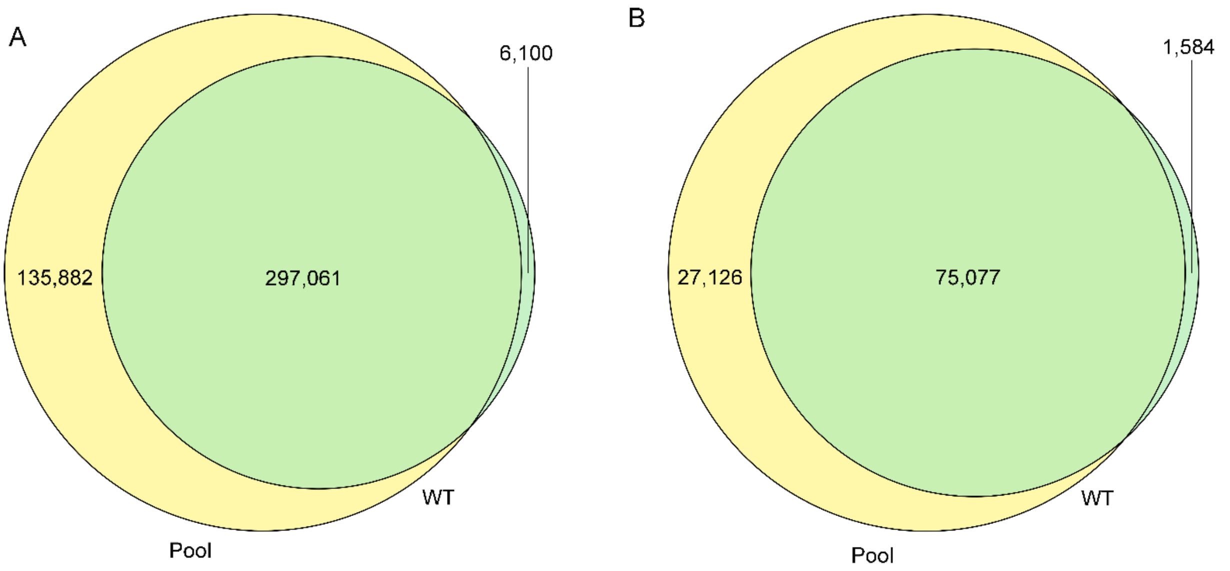

Supplementary Figure 1. Single nucleotide polymorphisms (SNPs) and insertion deletions (InDels) polymorphisms between WT and the pool of *bos1-1* mutant. **(A)** Differential SNPs between WT and the pool of *bos1-1* mutant. **(B)** Differential InDels between WT and the pool of *bos1-1* mutant.

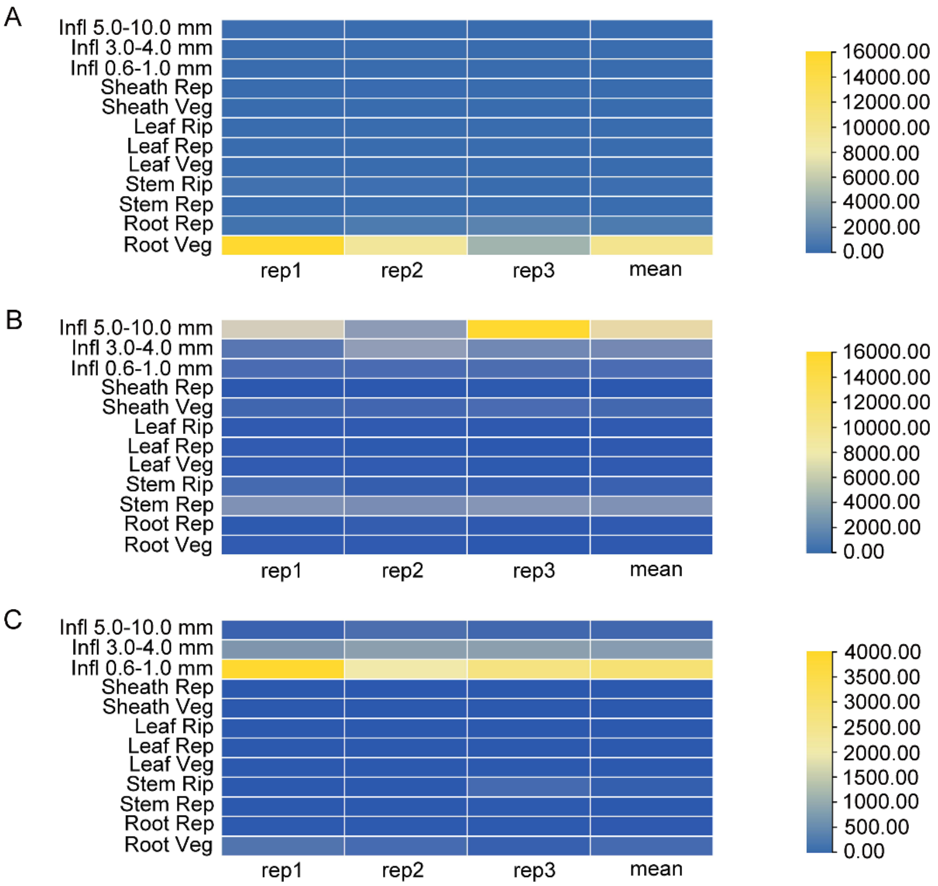

Supplementary Figure 2. Spatial-temporal of *OsPIN2* **(A)**, *OsPIN3* **(B)**, *and FZP* **(C)** gene expression in various tissues /organ throughout entire growth in the field. Three biological replications were performed.
